# Association between transabdominal uterine artery Doppler and small-for-gestational-age: a systematic review and meta-analysis

**DOI:** 10.1186/s12884-023-05968-w

**Published:** 2023-09-13

**Authors:** Ruijuan Zhi, Xiangping Tao, Qingtao Li, Ming Yu, Honge Li

**Affiliations:** 1 Department of Ultrasound, The First People’s Hospital of Lianyungang, No.6 Zhenhua East Road, Haizhou District, Lianyungang, 222061 P.R. China; 2 Department of Pediatrics, The Affiliated Lianyungang Oriental Hospital of Xuzhou Medical University, Lianyungang, 222000 P.R. China; 3 Department of Obstetrics and Gynecology, The First People’s Hospital of Lianyungang, Lianyungang, 222061 P.R. China

**Keywords:** Uterine-artery doppler, Small for gestational age, Meta-analysis, Pulsatility index, Resistance index

## Abstract

**Background:**

The association between uterine artery Doppler (UtA) measurements and small for gestational age (SGA) has not been quantitatively analyzed throughout the whole pregnancy. This systematic review and meta-analysis aims to comprehensively explore the association between UtA measurements and SGA in the first, second, and third trimesters.

**Methods:**

Studies were searched from Pubmed, Embase, Cochrane Library, and Web of Science. Weighted mean difference (WMD), odds ratio (OR), and relative risk (RR) with 95% confidence interval (CI) were used as the effect size. Heterogeneity of all effect sizes was tested and quantified using I^2^ statistics. Sensitivity analysis was conducted for all outcomes, and publication bias was evaluated using Begg’s test.

**Results:**

A total of 41 studies were finally included in our meta-analysis. In the first trimester, mean PI was significantly higher in the SGA group than the non-SGA group (WMD: 0.31, 95%CI: 0.19–0.44). In the second trimester, odds of notch presence (OR: 2.54, 95%CI: 2.10–3.08), mean PI (WMD: 0.21, 95%CI: 0.12–0.30), and mean RI (WMD: 0.05, 95%CI: 0.05–0.06) were higher in the SGA group. Also, abnormal UtA measurements were associated with the increased odds of SGA (all *P* < 0.05). In the third trimester, PI z-score (WMD: 0.62, 95%CI: 0.33–0.91) and PI MoM (WMD: 0.08, 95%CI: 0.06–0.09) showed a significant increase in the SGA group. The odds of SGA were higher in the women with mean PI > 95% (OR: 6.03, 95%CI: 3.24–11.24).

**Conclusions:**

Abnormal UtA measurements were associated with high odds of SGA, suggesting that UtA might be an adjunctive screening method for SGA in the whole pregnancy.

**Supplementary Information:**

The online version contains supplementary material available at 10.1186/s12884-023-05968-w.

## Background

Small for gestational age (SGA) refers to the fetal birth weight ≤ 10th percentile according to local standards [1]. Fetuses with late-onset SGA have a high risk of adverse perinatal outcomes, such as high rate of surgical delivery, low Apgar and arterial cord blood pH values, and high frequency of neonatal unit (NNU) admission [1]. Screening for SGA is a key element of prenatal care [2].

Uterine artery Doppler (UtA) has been used to assess the risk of SGA in pregnant women [3]. Studies on the association between UtA and the risk of SGA have been previously reported. He et al. have found that mean UtA-pulsatility index (UtA-PI) and UtA-resistance index (UtA-RI) were higher in the SGA fetuses compared to non-SGA fetuses [4]. Običan et al. have reported that abnormal UtA indices were significantly correlated with an increased risk of SGA [5]. Left uterine artery notching and PI > 95th percentile increased 1.76-fold and 1.83-fold risk of SGA, respectively [5]. However, several limitations existed in the separate original studies, including insufficient sample size or being limited to one region.

Meta-analysis is a powerful tool to combine results from two or more separate studies, which shows a good evidence strength and facilitates healthcare decision-making [6, 7]. A systematic review by Meler et al. have already reported the association between UtA and SGA, while the results are not quantitatively analyzed [8]. Cnossen et al. performed a meta-analysis to explore the predictive accuracy of UtA for SGA in the first and second trimesters, while they did not focus on the third trimester [9]. The persistent increase in the uterine artery impedance in the third trimesters increased the risk of SGA [8]. UtA examination can be conducted in transvaginal and transabdominal approaches, and transabdominal approach is recommended because most of the studies evaluating the UtA in the third trimester used a transabdominal approach [10]. Therefore, we performed a systematic review and meta-analysis based on the previously published studies to comprehensively explore the association between transabdominal UtA measurements and the risk of SGA in the first, second, and third trimesters.

## Methods

### Literature search strategy

This meta-analysis was performed based on the Preferred Reporting Items for Systemic Reviews and Meta-Analyses (PRISMA) guideline [11]. Pubmed, Embase, Cochrane Library, and Web of Science were searched by two researchers (RJZ and XPT) for relevant studies up to July 28, 2022. The search terms used were “Uterine Artery” OR “Arteries, Uterine” OR “Artery, Uterine” OR “Uterine Arteries” AND “Ultrasonography, Doppler” OR “Doppler Ultrasound” OR “Doppler Ultrasounds” OR “Ultrasound, Doppler” OR “Ultrasounds, Doppler” OR “Doppler Ultrasonography” OR “Doppler Ultrasound Imaging” OR “Doppler Ultrasound Imagings” OR “Imaging, Doppler Ultrasound” OR “Imagings, Doppler Ultrasound” OR “Ultrasound Imaging, Doppler” OR “Ultrasound Imagings, Doppler” OR “PI” OR “pulsatility index” OR “RI” OR “resistance index” OR “blood flow index” OR “diastolic notch” OR “blood flow score” OR “ratio of systolic and diastolic blood flow velocity” OR “the ratio of systolic peak value and end diastolic velocity of blood flow” OR “S/D” OR “systolic maximum flow velocity” OR “Systolic low velocity” OR “diastolic minimum flow velocity” OR “Diastolic flow velocity” AND “Infant, Small for Gestational Age” OR “Small for Gestational Age” OR “SGA” OR “Fetal Growth Retardation” OR “Intrauterine Growth Retardation” OR “Growth Retardation, Intrauterine” OR “Intrauterine Growth Restriction” OR “Fetal Growth Restriction”. We have registered this systematic review and meta-analysis with PROSPERO (registration number: CRD42023447101).

### Inclusion and exclusion criteria

Only studies meeting the following criteria were included: (1) patients: women with single pregnancy; (2) intervention and control: abnormal UtA group vs. normal UtA group or SGA group vs. non-SGA group; (3) outcome: SGA; (4) study design: case–control studies and cohort studies.

The UtA parameters we observed in this study were mean UtA-PI, mean UtA-RI, multiple of median (MoM) values of UtA-PI (UtA-PI MoM), UtA-PI z-score, and notch presence. PI was calculated as (peak systolic velocity-end diastolic velocity)/average velocity, and RI was calculated as (peak systolic velocity-end diastolic velocity)/peak systolic velocity. Mean PI and RI were the average from the left and right uterine arteries [12]. Notch presence meant the unilateral or bilateral notch in the diastolic notch [13]. Abnormal UtA includes: the presence of diastolic notch, high RI (RI > 95%, RI > 75%, RI > 90%), or high PI (PI > 95%) [13–16]. SGA was defined as the fetal birth weight ≤ 10th percentile according to local standards [1].

The following exclusion criteria were adopted: (1) animal studies; (2) studies irrelevant to the topic (studies not on transabdominal UtA or SGA definition not conformed); (3) reviews, meta-analyses, case reports, protocols, conference abstracts, guidelines, and expert consensus; (4) not published in English.

### Data extraction

Two researchers (RJZ and XPT) independently evaluated the data suitable for this meta-analysis, and extracted the following information: the first author, publication year, country, study design, group, sample size, age, body mass index (BMI), birth weight, gestational age, complications, smoking, and Doppler time. If conflicts existed, a third researcher (HEL) provided the consultation.

### Methodological quality appraisal

The quality of case–control studies and cohort studies was assessed using Newcastle–Ottawa Scale (NOS) [17]. For case–control studies, three items (selection, comparability, and exposure) were assessed. For cohort studies, three items (selection, comparability, and outcome) were evaluated. The total score of this scale was 9 points, and study quality was regarded as poor (0–3 points), fair (4–6 points), and good (7–9 points).

### Statistical analysis

Weighted mean difference (WMD) with 95% confidence interval (CI) was used as the effect size for measurement data, and odds ratio (OR) with 95%CI was used as effect size for counting data. If relative risk (RR) was provided in the publications, RR was combined for analysis. Heterogeneity was tested for all effect sizes and quantified using the I^2^ statistics. If the heterogeneity statistic I^2^ ≥ 50%, random effect model was used for analysis; otherwise, fixed effect model was used for analysis. Sensitivity analysis was performed to assess the effect of a single study on the whole estimate by removing studies one by one. Publication bias was assessed using Begg’s test for the outcomes included in more than nine studies [18]. All statistical analysis was performed using Stata15.1 software (StataCorp, College Station, TX, USA), and* P* < 0.05 was considered to be statistically significant.

### Certainty of evidence

The certainty of the evidence was assessed using the Grading of Recommendations Assessment, Development, and Evaluation (GRADE). The GRADE system categorized the certainty of the pooled estimate of effect as high, moderate, low, or very low according to the following criteria: study design, risk of bias, inconsistency, indirectness, imprecision, and other considerations. An evidence profile was produced to summarize the results using the GRADEpro GDT (https://gdt.gradepro.org/).

## Results

### Study selection and study characteristics

**Fig. 1:**
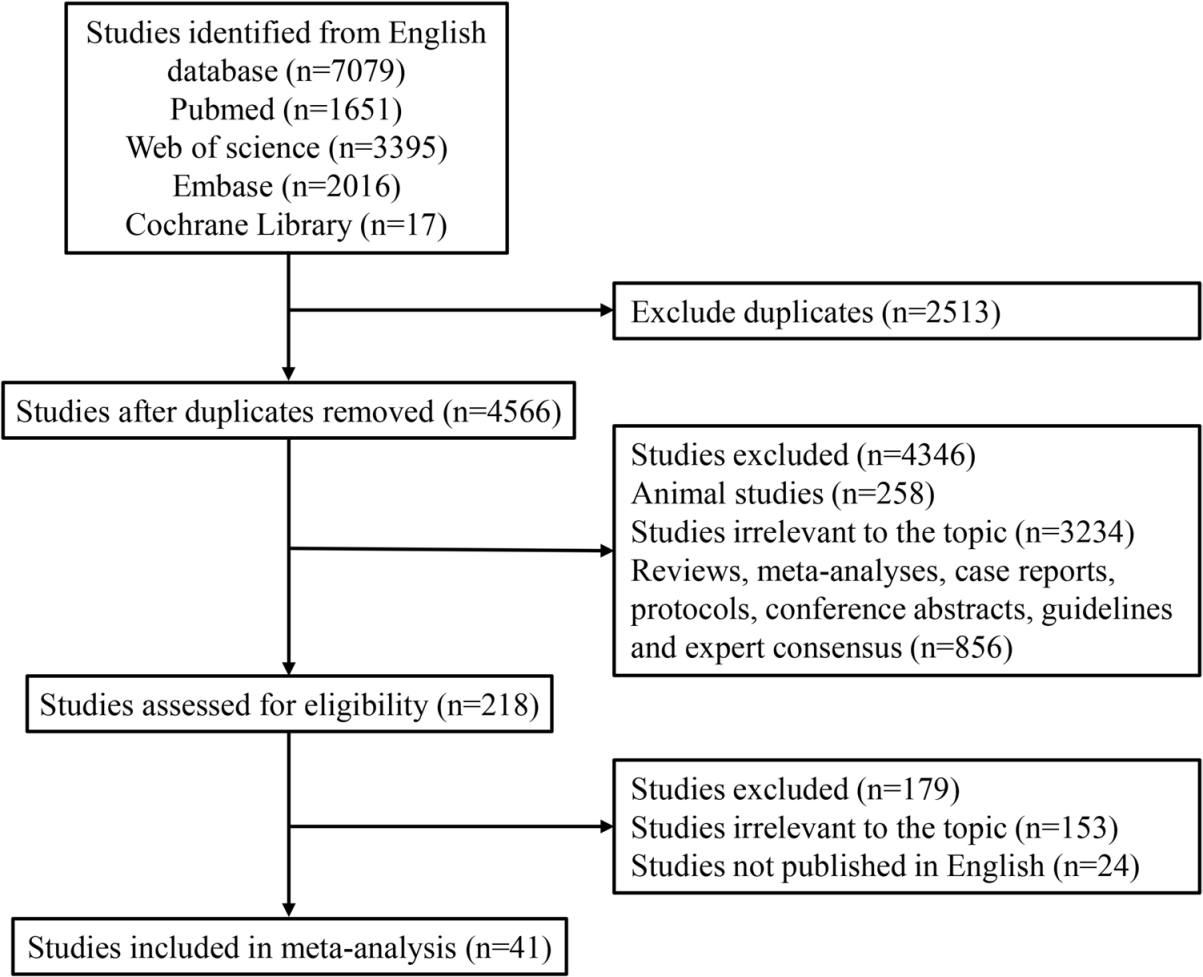
The flowchart of selecting studies

A total of 7,079 studies were identified from the above-mentioned databases. After removing 2,513 duplicates, 4,566 studies remained. After screening title and abstract, animal studies (*n* = 258), studies irrelevant to the topic (*n* = 3,234), and reviews, meta-analyses, case reports, protocols, conference abstracts, guidelines and expert consensus (*n* = 856) were eliminated. After screening the full texts, 179 studies were removed due to irrelevant to the topic (*n* = 153) or not published in English (*n* = 24). Finally, 41 studies were included in our meta-analysis (Fig. 1) [1, 4, 5, 12–16, 19–51]. Table 1 displays the characteristics of the included studies. There were 38 cohort studies and 3 case–control studies. According to the Newcastle Ottawa scale, 32 studies were assessed as good quality and 9 studies were assessed as fair quality (Supplementary table S1-S2).

**Table 1:** Characteristics of included studies

Author	Year	Country	Study design	Group	Sample size	Age (years)	BMI (kg/m2)	Birth weight (g)	GA (weeks)	Complications	Smoking (number)	Doppler time (weeks)	QA
Arakaki	2020	Japan	Cohort	SGA (early-onset)	28	34 (20, 43)^a^	19.9 (16.3, 25.0)^a^	2298 (805, 2637)^a^	38w6d (28w4d, 40w5d)^a^	PIH	NA	11–13 weeks	7
				SGA (late-onset)	73	34 (22, 44)^a^	19.6 (15.8, 27.7)^a^	2493 (1703, 2943)^a^	39w4d (33w6d, 41w4d)^a^	PIH			
				Non-SGA	1261	34 (18, 52)^a^	20.1 (15.4, 43.8)^a^	3032 (737, 4483)^a^	39w2d (24w4d, 41w6d)^a^	PIH			
Arrue	2017	Spain	Cohort	Normal UtA	385	31.6 ± 4.6	23.03 ± 3.7	3293.8 ± 431.0	39.5 ± 1.4	NA	NA	Third trimester	8
				Abnormal UtA	56	32.7 ± 3.9	24.4 ± 4.5	3033.37 ± 689.7	38.5 ± 2.2				
Borna	2019	Iran	Cohort	SGA	11	28(17, 43)^a^	NA	3069.88 (1200, 4100)^a^	NA	Severe hypertension, non-severe hypertension	NA	18–22 weeks	6
				Non-SGA	97								
Carter	2015	USA	Cohort	Abnormal UtA	1192	31.5 (17–49)^a^	26.4 (15.50, 67.67)^a^	NA	NA	Preeclampsia, early preeclampsia, chronic hypertension, pregestational diabetes mellitus	NA	11–14 weeks	7
				Normal UtA									
Ciobanu	2019	UK	Cohort	SGA	1012	31.7 (27.2, 35.4)^b^	NA	NA	36.1 (35.9, 36.4)^b^	Chronic hypertension, diabetes mellitus type 1, diabetes mellitus type 2	NA	35–36 weeks	6
				Non-SGA	8592	32.2 (28.1, 35.7)^b^	NA	NA	36.1 (35.9, 36.4) ^b^	Chronic hypertension; diabetes mellitus type 1, diabetes mellitus type 2			
Drouin	2018	Canada	Cohort	SGA	486	28.9 (26.5, 31.5)^b^	23.2 (21.4, 26.1)^b^	2748 (2542, 2894)^b^	39.7 (38.9, 40.6)^b^	Chronic hypertension, diabetes mellitus; rheumatoid disease	NA	11–13 weeks	6
				Non-SGA	4149	28.7 (26.1, 31.3)^b^	23.8 (21.7, 27.1)^b^	3368 (3118, 3644)^b^	39.9 (39.0, 40.7)^b^	chronic hypertension, diabetes mellitus, rheumatoid disease			
Dugoff	2005	USA	Cohort	Abnormal UtA	1008	33 ± 5	23.3 ± 4.0	NA	38.8 ± 2	NA	61	10–14 weeks	7
				Normal UtA									
El-Hamedi	2005	UK	Cohort	Abnormal UtA	98	NA	NA	NA	NA	NA	NA	Second trimester	5
				Normal UtA	232	NA	NA	NA	NA	NA			
Espinoza	2010	USA	Cohort	SGA	396	24 (15, 46)^a^	NA	2770 (550, 3070)^a^	39.3 (27.4, 41.9)^a^	Preeclampsia	55	23–25 weeks	8
				Non-SGA	3153	26 (13, 46)^a^	NA	3450 (870, 5350)^a^	39.6 (25.6, 43)^a^	Preeclampsia	334		
Ghi	2010	Italy	Cohort	Abnormal UtA	62	33.67 ± 5.34	21.66 ± 1.70	2408 ± 763	36.20 ± 3.45	NA	NA	26–28 weeks	6
				Normal UtA	42	32.64 ± 5.75	21.85 ± 1.97	3116 ± 463	38.59 ± 1.76	NA			
González-González	2017	Spain	Cohort	SGA	193	30.45 ± 6.37	26.4 ± 5.72	2421.1 ± 529.6	38.8 ± 3.3	Pregestational diabetes, chronic hypertension	64	11–13 weeks	7
				Non-SGA	795	31.11 ± 5.78	26.3 ± 5.29	3318.4 ± 520.9	39.5 ± 1.9	Pregestational diabetes, chronic hypertension	150		
Groom	2009	New Zealand	Cohort	Normal UtA	2189	28.4 ± 5.7	25.2 ± 5.2	3403 ± 561	39.9 ± 1.9	NA	219 (at 15-wk visit); 227 (in pregnancy)	20–24 weeks	6
				Normal + abnormal UtA									
				Abnormal + normal UtA									
Hafner	2006	Austria	Cohort	SGA	2489	29.2 ± 5.2	23.7 ± 4.6	NA	NA	NA	NA	21–23 weeks	9
				Non-SGA									
He	2021	China	Cohort	SGA	76	29.6 ± 3.4	22.8 ± 1.5	1830 (920–2990)^a^	34.0 (28.0–39.1)^a^	Gestational diabetes mellitus, preeclampsia	NA	11–13 weeks	9
				Non-SGA	1720	30.5 ± 3.9	24.7 ± 1.7	3300 (2370–4640)^a^	39.0 (36.0–41.0)^a^	Gestational diabetes mellitus, preeclampsia			
Hershkovitz	2005	UK	Cohort	Normal UtA	55	NA	NA	3080(828, 4460)^a^	39 (27, 42)^a^	Preeclampsia	NA	20–24 weeks	7
				Abnormal UtA (unilateral)	11	NA	NA	2300(1282, 3820)^a^	37 (32, 39.5)^a^	Preeclampsia	NA		
				Abnormal UtA (bilateral)	22	NA	NA	1892(828, 3610)^a^	35 (27, 40)^a^	Preeclampsia	NA		
Kienast	2016	Germany	Cohort	SGA	40	25.8 ± 7.1	23 ± 1.7	2288.6 ± 479.1	36.6 ± 3.8	Chronic hypertension, systemic lupus erythematosus	NA	18–25 weeks	9
				Non-SGA	306	24.4 ± 5.0	22.9 ± 2.4	3088.6 ± 404.0	38.6 ± 2.7	Chronic hypertension, Systemic lupus erythematosus	NA		
Konchak	1995	USA	Cohort	Normal UtA	103	27.1 ± 5.1	NA	3086 ± 690.8	NA	NA	NA	17–22 weeks	7
				Abnormal UtA									
Lobmaier	2021	Germany	Cohort	SGA	149	32.1 ± 4.5	22.5 ± 3.3	2630 ± 360	39.9 ± 1.2	PIH/PE, autoimmune disease, history of SGA	12	Third trimester	7
				Non-SGA	143	32.2 ± 4.3	22.0 ± 2.7	3488 ± 369	38.7 ± 1.3	PIH/PE, autoimmune disease, history of SGA	4		
Maged	2017	Egypt	Cohort	Non-SGA	297	27.37 ± 3.66	24.49 ± 2.04	3277.07 ± 243.06	38.84 ± 1.98	NA	41	18–22 weeks	8
				SGA	52	28.54 ± 3.05	24.5 ± 2.15	2466.54 ± 306.1	37.35 ± 1.47		18		
Maroni	2011	Italy	Cohort	Abnormal UtA	66	34.42 ± 4.44	23.81 ± 2.82	2942 ± 583	38.2 ± 1.64	Late-onset pre-eclampsia	NA	34 weeks	7
				Normal UtA	66	34.12 ± 4.12	23.36 ± 2.38	3404 ± 469	38.9 ± 1.3	Late-onset pre-eclampsia	NA		
McCowan	2010	New Zealand	Cohort	SGA	376	28.3 ± 5.9	26.2 ± 6.0	2573 ± 605	38.7 ± 3.8	NA	72	20 weeks	9
				Non-SGA	3137	28.1 ± 5.8	25.4 ± 5.1	3487 ± 514	39.6 ± 2.0	NA	315		
Miranda	2017	Spain	Case–control	SGA	175	32 ± 5	22.4 ± 3.7	2421 ± 570	38.4 ± 2.8	Chronic hypertension, autoimmune disease, previous history of SGA	26	32–36 weeks	8
				Non-SGA	875	31 ± 5	22.8 ± 4.1	3381 ± 396	39.7 ± 1.3	Chronic hypertension, autoimmune disease, previous history of SGA	75		
Mitsui	2016	Japan	Cohort	Abnormal UtA	24	33.3 ± 6.2	24.3 ± 5.8	2450.4 ± 768.1	37.6 ± 3.1	PIH	NA	Second trimester	7
				Normal UtA	13	34.3 ± 5.6	29.1 ± 6.4	2982.0 ± 576.6	39.3 ± 1.6	PIH	NA		
Miyakoshi	2001	Japan	Cohort	Abnormal UtA	28	32.6 ± 4.16	NA	2,934 ± 417.3	38.9 ± 1.8	PIH	NA	21–24 weeks	8
				Normal UtA	331								
Običan	2020	USA	Cohort	Abnormal UtA	200	27 (23, 31) ^b^	25 (20.9, 31.4) ^b^	NA	NA	Chronic hypertension,	20	Third trimester	6
				Normal UtA									
Ohkuchi	2000	Japan	Cohort	SGA	15	28.1 ± 3.7	NA	2438 ± 276	39.6 ± 1.5	NA	NA	16–23 weeks	8
				Non-SGA	264	28.7 ± 4.0	NA	3105 ± 744	39.1 ± 2.2	NA	NA		
Paules	2019	Spain	Case–control	Non-SGA	202	33.6 (30.5, 36.5)^b^	22.8 (20.5, 25.3)^b^	3020 (2510, 3420) ^b^	39.0 (35.7, 40.1) ^b^	Chronic hypertension, pregestational diabetes	23	35–37 weeks	7
				SGA	184	33.4 (29.0, 36.4)^b^	21.6 (19.8, 23.4)^b^	2337 (1935, 2613)^b^	37.7 (37.0, 39.4)^b^	Chronic hypertension, pregestational diabetes	48		
Phupong	2003	Thailand	Cohort	Abnormal UtA	58	26.4 ± 4.8	NA	NA	NA	preeclampsia	NA	22–28 weeks	5
				Normal UtA	264								
Quant	2016	USA	Cohort	SGA	40	31 (27–35)^b^	26.0 ± 7.01	NA	NA	Chronic hypertension	NA	18–24 weeks	7
				Non-SGA	333								
Rial-Crestelo	2019	Spain	Cohort	SGA	155	34 ± 5.1	23 ± 3.6	2733 ± 250	40 ± 1.3	Maternal disease, previous FGR	25	32–34 weeks	9
				Non-SGA	875	34 ± 5.4	24 ± 4	3423 ± 384	40 ± 1.3	Maternal disease, previous FGR	79		
Rodríguez	2018	Chile	Cohort	Abnormal UtA	33	28.2 ± 7.47	34.5 ± 5.02	2533 ± 561.3	36.4 (35.7, 37.7)^b^	Chronic hypertension, systemic lupus erythematosus, diabetes mellitus type-2	0 (0)	34 weeks	7
				Normal UtA	53	26.0 ± 7.04	32.7 ± 5.47	3227 ± 447.9	38 (37.0, 38.1)^b^	Chronic hypertension, systemic lupus erythematosus, diabetes mellitus type-2	3		
Roeder	2014	USA	Cohort	Normal UtA	108	32.2 ± 5.3	24.2 (22.1, 27.8)^b^	NA	NA	Prior preeclampsia, chronic hypertension, prior IUGR, pregestational diabetes	NA	24–26 weeks	6
				Abnormal UtA	24								
Rueangjaroen	2021	Thailand	Cohort	SGA	24	29.5 ± 5.2	23.4 ± 4.3	2256 ± 551	37.3 ± 2.8	NA	NA	11–14 weeks; 18–22 weeks	8
				Non-SGA	311	29.7 ± 5.1	23.1 ± 4.5	3076 ± 455	38.2 ± 2.1	NA	NA		
Schwartz	2014	USA	Cohort	SGA	56	29.6 ± 5.8	26.2 ± 7.7	NA	NA	Chronic hypertension	9	11–14 weeks	9
				Non-SGA	522	30.8 ± 5.8	27.1 ± 6.8	NA	NA	Chronic hypertension	48		
Seravalli	2014	USA	Cohort	SGA	172	28 (22, 35)^b^	27.5 (23.5, 34.4)^b^	NA	NA	History of diabetes, history of preeclampsia, history of hypertension, prior IUGR	NA	18–22 weeks	7
				Non-SGA	1810	30 (24, 35)^b^	27.6 (24.2, 32.6)^b^	NA	NA	history of diabetes, history of preeclampsia, history of hypertension, prior IUGR	NA		
Shwarzman	2013	Israel	Cohort	Normal UtA	144	29.35 ± 5.8	NA	NA	34.33 ± 2.99	Diabetes mellitus, hypertensive disorders	NA	34–37 weeks	8
				Abnormal UtA (unilateral pathologic waveforms)	37	27 .30 ± 6.44	NA	NA	34.08 ± 2.80	Diabetes mellitus, hypertensive disorders	NA		
				Abnormal UtA (bilateral pathologic waveforms)	17	30.12 ± 5.3	NA	NA	34.33 ± 3.70	Diabetes mellitus, hypertensive disorders	NA		
Triunfo	2017	Spain	Case–control	SGA	46	32.4 ± 4.7	23.9 ± 3.1	2215.1 ± 576.9	37.4 ± 3.1	Chronic hypertension, gestational diabetes, autoimmune disease, coagulation disorders, neurological disorders, endocrinological disorders	23 (< 10 cigarettes/day); 12 (≥ 10 cigarettes/day)	First trimester; Second trimester; Third trimester	8
				Non-SGA	92	31.7 ± 4.8	24.0 ± 3.5	3374.2 ± 404.2	39.7 ± 1.2	Chronic hypertension, gestational diabetes, autoimmune disease, coagulation disorders, neurological disorders, endocrinological disorders	< 10 cigarettes/day: 8(8.7%);≥ 10 cigarettes/day: 6(6.5%)		
Valiño	2016	UK	Cohort	SGA	379	37.7 (26.9, 35.3)^b^	NA	NA	40.0 (39.1, 40.9)^b^	Chronic hypertension, diabetes mellitus, SLE/APS	365	35–37 weeks	8
				Non-SGA	3509								
Ventura	2015	Peru	Cohort	Abnormal UtA	91	33 (28, 36)^b^	NA	2840 (2370, 3320)^b^	39 (37, 40)^b^	Hypertension, diabetes mellitus	32	28 weeks	8
				Normal UtA	174			3090 (2795, 3400)^b^	39 (38, 40)^b^				
Viola	2014	USA	Cohort	SGA	191	28 (22, 35)^b^	26.1 (22.9, 33.7)^b^	2605 (2281, 2736)^b^	39 (37.4, 40)^b^	Diabetes, hypertension, prior preeclampsia, prior IUGR	28	11–14 weeks	8
				Non-SGA	2076	30 (25, 35)^b^	26.6 (23.1, 32)^b^	3320 (3045, 3604)^b^	39.3 (38.3, 40)^b^	Diabetes, hypertension, prior preeclampsia, prior IUGR	192		
Zarean	2018	Iran	Cohort	Normal UtA	60	30.53 ± 5.51	NA	3236.50 ± 592.64	37.93 ± 1.96	Preeclampsia, gestational diabetes, hypertension, hypertension with preeclampsia	NA	30–34 weeks	7
				Abnormal UtA	40	28.50 ± 6.03	NA	2422.75 ± 473.95	36.49 ± 2.51	Preeclampsia, gestational diabetes, hypertension, hypertension with preeclampsia	NA		

*Abbreviation*: *BMI* body mass index, *GA* gestational age, *QA* quality assessment, *SGA* small-for-gestational age, *PIH* pregnancy-induced hypertension, *NA* not applicable, *UtA* uterine artery Doppler, *GH* gestational hypertension, *PE* pre-eclampsia

^a^ Data are presented as the median (range)

^b^Data are presented as the median (IQR)

### Comparison of UtA measurements between SGA group and non-SGA group

**Fig. 2:**
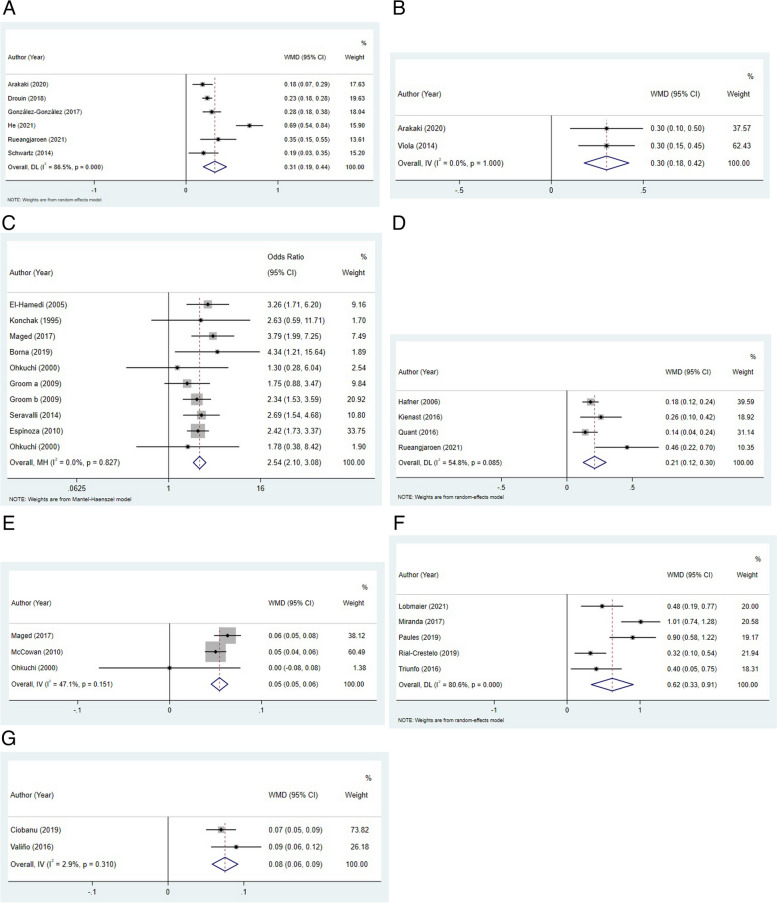
Forest plots regarding to mean PI (**A**) and PI Z-score (**B**) in the first trimester; notch presence (**C**), mean PI (**D**), and mean RI (**E**) in the second trimester; PI z-score (**F**) and PI MoM (**G**) in the third trimester

In the first trimester, mean PI and PI z-score were significantly higher in the SGA group than in the non-SGA group (WMD: 0.31, 95%CI: 0.19–0.44; WMD: 0.30, 95%CI: 0.18–0.42) (Fig. 2A-B). There was no significant difference in mean RI between the SGA group and non-SGA group (WMD: 0.06, 95%CI: -0.04–0.16). A study by Arakaki et al. reported that RI z-score was significantly higher in the SGA group compared to non-SGA group [12]. In the second-trimester, we found that the risk of notch presence in the SGA group was higher than in the non-SGA group (OR: 2.54, 95%CI: 2.10–3.08) (Fig. 2C). Compared to non-SGA group, SGA group showed higher values of mean PI (WMD: 0.21, 95%CI: 0.12–0.30) (Fig. 2D) and mean RI (WMD: 0.05, 95%CI: 0.05–0.06) (Fig. 2E). Seravalli et al. reported that PI z-score of SGA group was higher than that of non-SGA group [46]. Espinoza et al. had reported the risk of PI > 95% and RI > 95% in SGA group was higher than in the non-SGA group [24]. In the third-trimester, PI z-score and PI MoM showed a significant increase in the SGA group compared with non-SGA group, with WMD value of 0.62 (95%CI: 0.33–0.91) (Fig. 2F) and 0.08 (95%CI: 0.06–0.09) (Fig. 2G), respectively. Rial-Crestelo et al. reported that SGA group showed a higher risk of PI > 95% compared to non-SGA group [41]. The results were summarized in Table 2.

**Table 2:** Comparison of UtA parameters between SGA groups and non-SGA groups

Outcomes	Number of studies	Number of participants	WMD/OR (95%CI)	*P*	I^2^
**The first trimester**					
Mean RI	2	3158	0.06 (-0.04, 0.16) ^a^	0.233	97.40%
Sensitivity analysis			0.06 (-0.04, 0.16)		
Mean PI	6	9694	0.31 (0.19, 0.44) ^a^	< 0.001	86.50%
Sensitivity analysis			0.31 (0.19, 0.44)		
PI Z-score	2	3629	0.30 (0.18, 0.42) ^a^	< 0.001	0.00%
Sensitivity analysis			0.30 (0.18, 0.42)		
**The second trimester**					
Notch presence	9	10974	2.54 (2.10, 3.08) ^b^	< 0.001	0.00%
Sensitivity analysis			2.54 (2.10, 3.08)		
Publication bias			Z = 0.36	0.974	
Mean PI	4	3543	0.21 (0.12, 0.30) ^a^	< 0.001	54.80%
Sensitivity analysis			0.21 (0.12, 0.30)		
Mean RI	3	4141	0.05 (0.05, 0.06) ^a^	< 0.001	47.10%
Sensitivity analysis			0.05 (0.05, 0.06)		
**The third trimester**					
PI z-score	5	2918	0.62 (0.33, 0.91) ^a^	< 0.001	80.60%
Sensitivity analysis			0.62 (0.33, 0.91)		
PI MoM	2	13492	0.08 (0.06, 0.09) ^a^	< 0.001	2.90%
Sensitivity analysis			0.08 (0.06, 0.09)		

*Abbreviation*: *UtA* uterine artery Doppler, *SGA* small for gestational age, *PI* pulsatility index, *RI* resistance index, *MoM* multiple of median, *WMD* weighted mean difference, *RR* relative risk, *OR* odds ratio, *CI* confidence interval

^a^ presented WMD

^b^ presented OR

### Comparison of SGA incidence between abnormal UtA group and normal UtA group

**Fig. 3:**
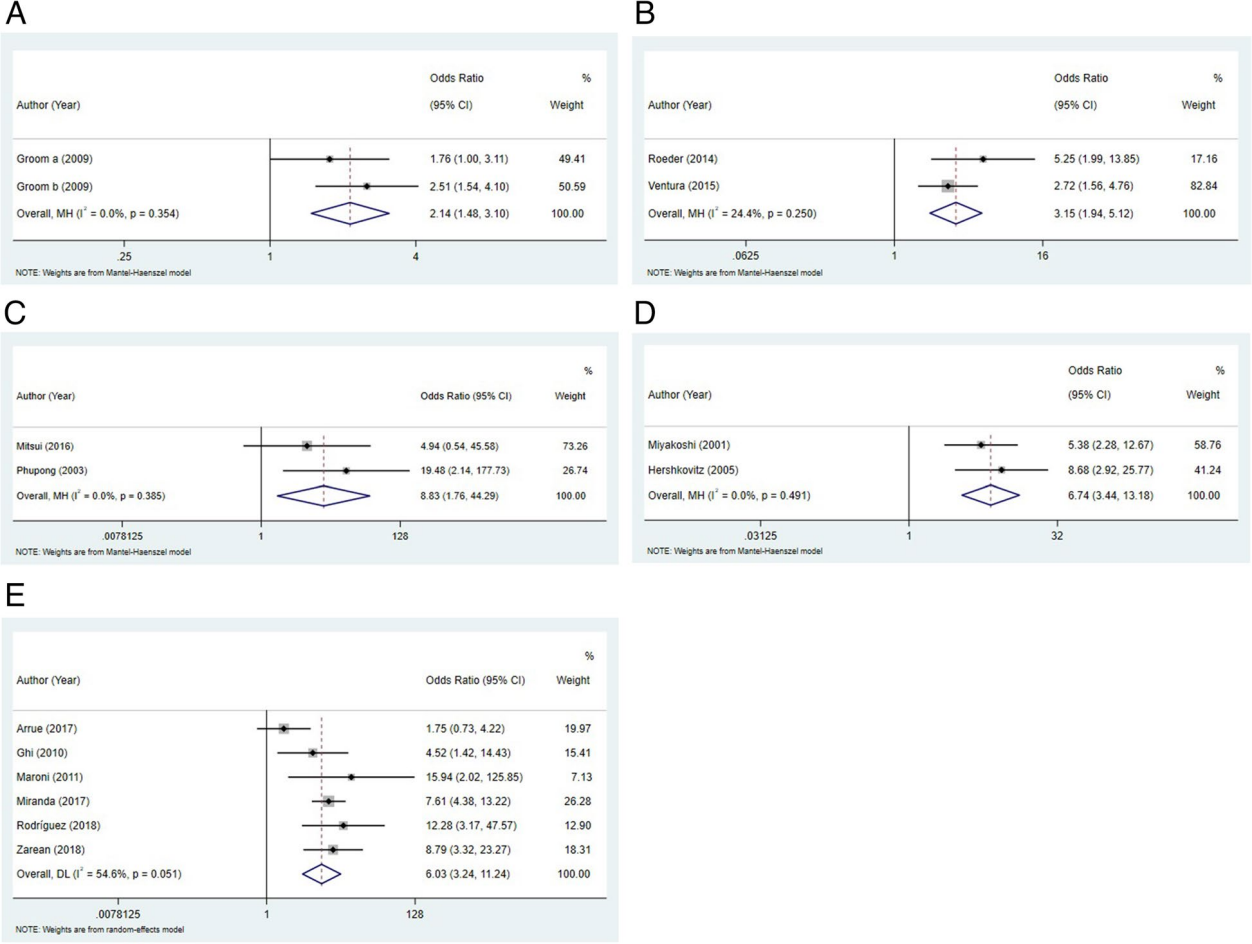
Forest plots regarding to mean RI > 90% (**A**), mean PI > 95% (**B**) notch presence (**C**), and mean PI > 95% or notch presence (**D**) in the second trimester; mean PI > 95% (**E**) in the third trimester

In the first trimester, there was no significant association between SGA and RI > 75% (RR: 2.61, 95%CI: 0.68–10.08) or RI > 95% (RR: 1.55, 95%CI: 0.73–3.26). Dugoff et al. reported that the risk of SGA incidence was higher in RI > 90% group compared to RI ≤ 90% group [22]. In the second trimester, we found that the odds of SGA incidence were significantly higher in women with mean RI > 90% (OR: 2.14, 95%CI: 1.48–3.10) (Fig. 3A), mean PI > 95% (OR: 3.15, 95%CI: 1.94–5.12) (Fig. 3B), notch presence (OR: 8.83, 95%CI: 1.76–44.29) (Fig. 3C), and mean PI > 95% or notch presence (OR: 6.74, 95%CI: 3.44–13.18) (Fig. 3D). In the third trimester, women with mean PI > 95% had higher odds of SGA than women with mean PI ≤ 95% (OR: 6.03, 95%CI: 3.24–11.24) (Fig. 3E). Običan et al. have found that the risk of SGA was higher in case of mean PI > 95% or notch presence [5]. The results were shown in Table 3.

**Table 3:** Comparison of SGA incidence between abnormal and normal UtA groups

SGA incidence	Number of studies	Number of participants	RR/OR (95%CI)	*P*	I^2^
**The first trimester**					
Mean RI > 75%	2	2200	2.61 (0.68, 10.08)^b^	0.163	79.10%
Sensitivity analysis			2.61 (0.68, 10.08)		
Mean RI > 95%	2	2200	1.55 (0.73, 3.26) ^b^	0.254	43.70%
Sensitivity analysis			1.55 (0.73, 3.26)		
**The second trimester**					
Mean RI > 90%	1	3968	2.14 (1.48, 3.10) ^a^	< 0.001	0.00%
Sensitivity analysis			2.14 (1.48, 3.10)		
Mean PI > 95%	2	397	3.15 (1.94, 5.12) ^a^	< 0.001	24.40%
Sensitivity analysis			3.15 (1.94, 5.12)		
Notch	2	359	8.83 (1.76, 44.29) ^a^	0.008	0.00%
Sensitivity analysis			8.83 (1.76, 44.29)		
Mean PI > 95% or Notch	2	447	6.74 (3.44, 13.18) ^a^	< 0.001	0.00%
Sensitivity analysis			6.74 (3.44, 13.18)		
**The third trimester**					
Mean PI > 95%	6	1913	6.03 (3.24, 11.24) ^a^	< 0.001	54.60%
Sensitivity analysis			6.03 (3.24, 11.24)		

*Abbreviation*: *UtA* uterine artery Doppler, *SGA* small for gestational age, *PI* pulsatility index, *RI* resistance index, *WMD* weighted mean difference, *RR* relative risk, *OR* odds ratio, *CI* confidence interval

^a^ presented OR

^b^ presented RR

### Sensitivity analysis and publication bias

Sensitivity analysis showed that no study displayed an important effect on the final pooled UtA measurements and SGA incidence (Tables 1 and 2). There was no evidence of publication bias in the reporting of notch presence in the second-trimester across studies (Z = 0.36, *P* = 0.974).

### Certainty of evidence

We used GRADE to assess the level of evidence. The results showed very low level of evidence for all outcomes (Supplementary table S3).

## Discussion

In this systematic review and meta-analysis, we explored the association between transabdominal UtA and SGA in the first, second, and third trimesters. The results showed that UtA measurements in the SGA group were significantly higher than the non-SGA group during the whole pregnancy. Also, SGA group had a higher odds of notch presence than the non-SGA group. In addition, we found that abnormal UtA was associated with the higher odds of SGA compared to normal UtA in the second and third trimesters.

Transabdominal UtA is a noninvasive test of the uteroplacental circulation, and has been applied to predict the risk of SGA in the clinical practice [4, 5, 44]. PI and RI are common observation indices in the UtA [52]. UtA-PI reflects total resistance distal to the measurement point, and UtA-RI reflects the vascular resistance at the measurement point [52]. A study showed a stable decrease in UtA-PI values until the late stages of pregnancy [53], whereas Cavoretto et al. found that UtA-PI showed a progressive non-linear decrease throughout the pregnancy by using fractional polynomial [54]. In this meta-analysis, we observed higher levels of UtA-PI and UtA-RI in women with SGA compared to those without SGA during the whole pregnancy. Previous studies have reported the similar findings [13, 31]. Borna et al. performed a study to identify patients at the risk of SGA using UtA, and they observed that mean UtA-PI in women with SGA newborns was significantly higher than those without SGA newborns [13]. Similarly, Maged et al. showed that UtA-RI was significantly higher in women who developed SGA compared to controls [31]. Also, we found that the odds of SGA were higher in the abnormal UtA group compared to normal UtA in the second and third trimesters. This finding was consisted with the studies by Običan et al. and Groom et al. [5, 15] Običan et al. suggested that the risk of SGA was significantly higher when PI > 95% [5]. Groom et al. indicated that the incidence of SGA was higher in women with UtA-RI > 90% than those with normal UtA-RI in the second trimester [15]. Evidence showed that trophoblastic invasion may be the reason for the increase of uterine vascular impedance; subsequently, changes in the uteroplacental circulation was detected by UtA [55, 56].

Diastolic notch is the characteristic of vessels with resistance, and depends on the compliance of vessel wall [57]. Dugoff et al. reported that 34.2% of 1067 American pregnant women had diastolic notches in the uterine artery; however, there was no significant association between diastolic notch and SGA [22]. He et al. also reported no significant association between notch and SGA although they found notching in the SGA fetuses was 40% higher than in the non-SGA fetuses [4]. One potential reason for this is that diastolic notch is dichotomous rather than numeric variables, which might introduce misclassification bias [4]. In this meta-analysis, we found a significant association between the notch presence and SGA and that notch presence was significantly associated with the increased odds of SGA. The similar finding was reported in former studies [5, 13, 35]. Borna et al. found that the incidence of SGA in women with notch was significantly greater than women without notch in ultrasonography [13]. In the study by Mitsui et al., a higher incidence of SGA was found in pregnant women with notch than those without (29.2% vs. 7.7%) [35]. This was consistent with the finding from the study of Običan et al. that UtA notch was significantly associated with SGA [5].

This meta-analysis explored the association between transabdominal UtA measurements and SGA in the first, second, and third trimesters, and found that abnormal UtA measurements were significantly associated with the high odds of SGA in the whole pregnancy. However, there are some limitations in this meta-analysis. First, judgement of normality or abnormality and classification of centiles in UtA measurements relies upon different curves and charts for uterine arteries, which may affect the reliability of the pooled results. In the future, a uniform judgement for abnormality needs to be explored. Second, there is heterogeneity in some results. Pregnancy complications (such as gestational hypertension, preeclampsia, gestational diabetes), maternal smoking, and history of SGA may be the sources of heterogeneity. However, we were unable to perform the subgroup analysis to explore the sources of heterogeneity because the above factors could not be analyzed based on the included studies. Third, SGA is defined as fetal birth weight ≤ 10th percentile of the standard weight of the fetus at the same gestational age. In included studies, standard weight varies from region to region, which may cause some bias on the results. Fourth, the method of conception of the included pregnancies is likely heterogeneous. UtA-PI values are significantly different in pregnancies after different conception method [58]. Fifth, due to the limitation of the included studies, we failed to explore the influence of the changes of UtA measurements on SGA in different pregnancy periods.

## Conclusion

In conclusion, our meta-analysis found a significant association between abnormal UtA measurements and increased odds of SGA in the whole pregnancy, indicating that UtA might be an adjunctive screening method for SGA in the whole pregnancy.

### Supplementary Information

**Additional file 1:** **Supplementary table S1.** Newcastle-Ottawa Scale assessment for cohort studies. **Supplementary table S2.** Newcastle-Ottawa Scale assessment for case-control studies. **Supplementary table S3.** Certainty of evidence for the included studies.

## Data Availability

The datasets used and/or analyzed during the current study are available from the corresponding author on reasonable request.

## Abbreviations

SGA: Small for gestational age
NNU: Neonatal unit
UtA: Uterine artery Doppler
PI: Pulsatility index
RI: Resistance index
PRISMA: Preferred Reporting Items for Systemic Reviews and Meta-Analyses
BMI: Body mass index
MoM: Multiple of median
WMD: Weighted mean difference
OR: Odds ratio
CI: Confidence interval
RR: Relative risk
