# Supplementary material for: Association between transabdominal uterine artery Doppler and small-for-gestational-age: a systematic review and meta-analysis
Source: BMC Pregnancy Childbirth. 2023 Sep 13;23:659. doi: 10.1186/s12884-023-05968-w (PMC10500919; doi:10.1186/s12884-023-05968-w)
Supplement: Supplementary file 1 — Additional file 1: Supplementary table S1. Newcastle-Ottawa Scale assessment for cohort studies. Supplementary table S2. Newcastle-Ottawa Scale assessment for case-control studies. Supplementary table S3. Certainty of evidence for the included studies. [file 12884_2023_5968_MOESM1_ESM.docx]

**Supplementary table S1 Newcastle-Ottawa Scale assessment for cohort studies**

| **Study ID** | **Selection** | | | | **Comparability** | **Outcome** | | | **Total score** |
| --- | --- | --- | --- | --- | --- | --- | --- | --- | --- |
|  | **Representativeness of exposed group** | **Representativeness of non-exposed group** | **Ascertainment of exposure** | **Demonstration that outcome was not present at start of study** | **Comparability of groups on the basis of design or analysis** | **Assessment of outcome** | **Follow up long enough** | **Adequacy of follow-up of groups** |  |
| Arakaki.2020 | * | * | * | * | * | * | - | * | 7 |
| Arrue.2017 | * | * | * | * | * | * | * | * | 8 |
| Borna.2019 | * | * | * | * | * | * | - | - | 6 |
| Carter.2015 | * | * | * | * | * | * | - | * | 7 |
| Ciobanu.2019 | * | * | * | * | * | * | - | - | 6 |
| Drouin.2018 | * | * | * | * | * | * | - | - | 6 |
| Dugoff.2005 | * | * | * | * | * | * | - | * | 7 |
| El-Hamedi.2005 | * | * | - | * | * | * | - | - | 5 |
| Espinoza.2010 | * | * | * | * | * | * | * | * | 8 |
| Ghi.2010 | * | * | * | * | * | * | - | - | 6 |
| González-González.2017 | * | * | * | * | * | * | - | * | 7 |
| Groom.2009 | * | * | * | * | * | * | - | - | 6 |
| Hafner.2006 | * | * | * | * | ** | * | * | * | 9 |
| He.2021 | * | * | * | * | ** | * | * | * | 9 |
| Hershkovitz.2005 | * | * | * | * | * | * | - | * | 7 |
| Kienast.2016 | * | * | * | * | ** | * | * | * | 9 |
| Konchak.1995 | * | * | * | * | * | * | - | * | 7 |
| Lobmaier.2021 | * | * | * | * | * | * | - | * | 7 |
| Maged.2017 | * | * | * | * | * | * | * | * | 8 |
| Maroni.2011 | * | * | * | * | * | * | - | * | 7 |
| McCowan.2010 | * | * | * | * | ** | * | * | * | 9 |
| Mitsui.2016 | * | * | * | * | * | * | - | * | 7 |
| Miyakoshi.2001 | * | * | * | * | * | * | * | * | 8 |
| Običan.2020 | * | * | * | * | * | * | - | - | 6 |
| Ohkuchi.2000 | * | * | * | * | * | * | * | * | 8 |
| Phupong.2003 | * | * | * | * | - | - | - | * | 5 |
| Quant.2016 | * | * | * | * | * | * | - | * | 7 |
| Rial-Crestelo.2019 | * | * | * | * | ** | * | * | * | 9 |
| Rodríguez.2018 | * | * | * | * | * | * | - | * | 7 |
| Roeder.2014 | * | * | * | * | * | * | - | - | 6 |
| Rueangjaroen.2021 | * | * | * | * | * | * | * | * | 8 |
| Schwartz.2014 | * | * | * | * | ** | * | * | * | 9 |
| Seravalli.2014 | * | * | * | * | * | * | - | * | 7 |
| Shwarzman.2013 | * | * | * | * | * | * | * | * | 8 |
| Valiño.2016 | * | * | * | * | * | * | * | * | 8 |
| Ventura.2015 | * | * | * | * | * | * | * | * | 8 |
| Viola.2014 | * | * | * | * | * | * | * | * | 8 |
| Zarean.2018 | * | * | * | * | * | * | - | * | 7 |

**Supplementary table S2 Newcastle-Ottawa Scale assessment for case-control studies**

| **Study ID** | **Selection** | | | | **Comparability** | **Exposure** | | | **Total score** |
| --- | --- | --- | --- | --- | --- | --- | --- | --- | --- |
|  | **Is the case definition adequate?** | **Representativeness of the cases** | **Selection of Controls** | **Definition of Controls** | **Comparability of cases and controls on the basis of the design or analysis** | **Ascertainment of exposure** | **Same method of ascertainment for cases and controls** | **Non-Response rate** |  |
| Miranda.2017 | * | * | * | * | ** | * | * | * | 8 |
| Paules.2019 | * | * | * | * | * | * | - | * | 7 |
| Triunfo.2017 | * | * | * | * | ** | * | * | * | 8 |

**Supplementary table S3 Certainty of evidence for the included studies**

| **Certainty assessment** | | | | | | | **Number of patients** | | **Effect** | | **Certainty** | **Importance** |
| --- | --- | --- | --- | --- | --- | --- | --- | --- | --- | --- | --- | --- |
| **Number of studies** | **Study design** | **Risk of bias** | **Inconsistency** | **Indirectness** | **Imprecision** | **Other considerations** |  |  | **Relative**  **(95% CI)** | **Absolute**  **(95% CI)** |  |  |
| **Mean RI (the first-trimester)** | | | | | | | | | | | | |
| 2 | observational studies | serious^a^ | serious^b^ | not serious | not serious | none | 177 | 2981 | - | MD 0.06 higher  (-0.04 lower to 0.16 higher) | ⨁◯◯◯  Very low | CRITICAL |
| **Mean PI (the first-trimester)** | | | | | | | | | | | | |
| 6 | observational studies | serious^a^ | serious^b^ | not serious | not serious | none | 936 | 8758 | - | MD 0.31 higher  (0.19 higher to 0.44 higher) | ⨁◯◯◯  Very low | CRITICAL |
| **PI Z-score (the first-trimester)** | | | | | | | | | | | | |
| 2 | observational studies | serious^a^ | not serious | not serious | not serious | none | 292 | 3337 | - | MD 0.30 higher  (0.178 higher to 0.422 higher) | ⨁◯◯◯  Very low | IMPORTANT |
| **Notch presence (the second-trimester)** | | | | | | | | | | | | |
| 9 | observational studies | serious^a^ | not serious | not serious | not serious | none | 164/1015 (16.2%) | 705/9959 (7.1%) | OR 2.544  (2.100 to 3.082) | 92 more per 1,000  (from 67 more to 119 more) | ⨁◯◯◯  Very low | CRITICAL |
| **Mean PI (the second-trimester)** | | | | | | | | | | | | |
| 4 | observational studies | serious^a^ | serious^b^ | not serious | not serious | none | 303 | 3240 | - | MD 0.212 higher  (0.124 higher to 0.3 higher) | ⨁◯◯◯  Very low | CRITICAL |
| **Mean RI (the second-trimester)** | | | | | | | | | | | | |
| 3 | observational studies | serious^a^ | not serious | not serious | not serious | none | 443 | 3698 | - | MD 0.054 higher  (0.045 higher to 0.063 higher) | ⨁◯◯◯  Very low | CRITICAL |
| **PI z-score (the third-trimester)** | | | | | | | | | | | | |
| 5 | observational studies | serious^a^ | serious^b^ | not serious | not serious | none | 743 | 2175 | - | MD 0.62 higher  (0.332 higher to 0.908 higher) | ⨁◯◯◯  Very low | IMPORTANT |
| **PI MoM (the third-trimester)** | | | | | | | | | | | | |
| 2 | observational studies | serious^a^ | not serious | not serious | not serious | none | 1391 | 12101 | - | MD 0.075 higher  (0.058 higher to 0.092 higher) | ⨁◯◯◯  Very low | IMPORTANT |
| **SGA incidence-Cut off: Mean RI > 75% (the first-trimester)** | | | | | | | | | | | | |
| 2 | observational studies | serious^a^ | serious^b^ | not serious | not serious | none |  |  | RR 2.613  (0.678 to 10.076) | 3 fewer per 1,000  (from 10 fewer to 1 fewer) | ⨁◯◯◯  Very low | NOT IMPORTANT |
| **SGA incidence-Cut off: Mean RI > 95% (the first-trimester)** | | | | | | | | | | | | |
| 2 | observational studies | serious^a^ | not serious | not serious | not serious | none |  |  | RR 1.545  (0.732 to 3.258) | 2 fewer per 1,000  (from 3 fewer to 1 fewer) | ⨁◯◯◯  Very low | NOT IMPORTANT |
| **SGA incidence-Cut off: Mean RI > 90% (the second-trimester)** | | | | | | | | | | | | |
| 1 | observational studies | serious^a^ | not serious | not serious | not serious | none | 37/246 (15.0%) | 284/3722 (7.6%) | OR 2.142  (1.480 to 3.101) | 74 more per 1,000  (from 33 more to 128 more) | ⨁◯◯◯  Very low | IMPORTANT |
| **SGA incidence-Cut off: Mean PI > 95% (the second-trimester)** | | | | | | | | | | | | |
| 2 | observational studies | serious^a^ | not serious | not serious | serious^c^ | none | 48/115 (41.7%) | 50/282 (17.7%) | OR 3.154  (1.942 to 5.123) | 227 more per 1,000  (from 118 more to 347 more) | ⨁◯◯◯  Very low | IMPORTANT |
| **SGA incidence-Notch (the second-trimester)** | | | | | | | | | | | | |
| 2 | observational studies | serious^a^ | not serious | not serious | very serious^c,d^ | none | 11/82 (13.4%) | 2/277 (0.7%) | OR 8.829  (1.760 to 44.293) | 53 more per 1,000  (from 5 more to 236 more) | ⨁◯◯◯  Very low | NOT IMPORTANT |
| **SGA incidence-Cut off: Mean PI > 95% or Notch (the second-trimester)** | | | | | | | | | | | | |
| 2 | observational studies | serious^a^ | not serious | not serious | serious^b^ | none | 27/61 (44.3%) | 37/386 (9.6%) | OR 6.738  (3.443 to 13.183) | 321 more per 1,000  (from 172 more to 487 more) | ⨁◯◯◯  Very low | IMPORTANT |
| **SGA incidence-Cut off: Mean PI > 95% (the third-trimester)** | | | | | | | | | | | | |
| 6 | observational studies | serious^a^ | serious^b^ | not serious | not serious | none | 109/432 (25.2%) | 70/1481 (4.7%) | OR 6.032  (3.236 to 11.241) | 183 more per 1,000  (from 91 more to 311 more) | ⨁◯◯◯  Very low | IMPORTANT |

CI, confidence interval; MD, mean difference; OR, odds ratio; RR, risk ratio.

Explanations: a, cohort study; b, I^2^ ≥ 50%; c, small sample size; d, 95% confidence interval is relatively large.
